# Increased Mortality Rate Associated with Chikungunya Epidemic, Ahmedabad, India

**DOI:** 10.3201/eid1403.070720

**Published:** 2008-03

**Authors:** Dileep Mavalankar, Priya Shastri, Tathagata Bandyopadhyay, Jeram Parmar, Karaikurichi V. Ramani

**Affiliations:** * Indian Institute of Management, Ahmedabad, India

**Keywords:** Chikungunya virus, excess mortality, causes of death, public health, disease outbreak, India, research

## Abstract

A total of 3,056 excess deaths epidemiologically linked to chikungunya occurred in 2006.

In 2005–2006, Réunion Island in the Indian Ocean reported ≈266,000 cases of chikungunya; 254 were fatal (case-fatality rate 1/1,000). India reported 1.39 million cases of chikungunya fever in 2006 with no attributable deaths; Ahmedabad, India, reported 60,777 suspected chikungunya cases. To assess the effect of this epidemic, mortality rates in 2006 were compared with those in 2002–2005 for Ahmedabad (population 3.8 million). A total of 2,944 excess deaths occurred during the chikungunya epidemic (August–November 2006) when compared with the average number of deaths in the same months during the previous 4 years. These excess deaths may be attributable to this epidemic. However, a hidden or unexplained cause of death is also possible. Public health authorities should thoroughly investigate this increase in deaths associated with this epidemic and implement measures to prevent further epidemics of chikungunya.

*Chikungunya virus*, an alphavirus of the family *Togaviridae*, is native to tropical Africa and Asia. This virus is transmitted to humans by mosquitoes. *Aedes aegypti* and *Ae*. *albopictus* are the 2 main vectors that transmit this disease (*1*). The first reported chikungunya outbreak occurred in Tanganyika (now Tanzania) in 1952–1953 (*2*). The word chikungunya is derived from the *Makonde* language in southeastern Tanzania and means “bent down or become contorted,” which indicates the classic posture the patient adopts because of severe joint pain. Symptoms of chikungunya include sudden onset of fever, severe arthralgia, and maculopapular rash. A specific symptom is severe incapacitating arthralgia, often persistent, which can result in long-lasting disability (*3*).

A major epidemic of this disease was reported in 2005–2006 in Réunion Island; ≈266,000 residents (34.3% of the population) of this Indian Ocean island were affected by chikungunya fever as of February 19, 2007. This epidemic also spread to France through imported cases from Réunion Island (*4*). Historically, chikungunya was considered self-limiting and nonfatal. However, 254 deaths on Réunion (case-fatality rate 1/1,000) that were attributed directly or indirectly to chikungunya during the epidemic changed this perspective (*1*,*4*).

India reported a massive chikungunya epidemic in 2006. Chikungunya has reemerged in India since 1973, when the attack rate was 37.5%. However, in the 2006 epidemic, the attack rate increased to 45% in some places (*4*). More than 1.39 million cases across 151 districts and 10 states were reported during this period (*5*). However, unlike the epidemic on Réunion Island, no deaths directly attributable to this disease were reported (*6*). The dominant vectors are *Ae*. *albopictus* on Réunion Island and *Ae*. *aegypti* in India (*4*). However, *Ae*. *albopictus* was also implicated in Kerala State, India (*7*).

Studies have indicated that the recent outbreak in the Indian Ocean islands was initiated by a strain related to East African isolates, from which viral variants have evolved with a traceable history of microevolution. This history could provide information for understanding the unusual magnitude and virulence of this chikungunya epidemic (*8*).

The purpose of this study was to analyze the association between the chikungunya epidemic in India and the mortality rate in the city of Ahmedabad. Such findings could show correlations between reported genomic mutations in chikungunya virus and its increased virulence. Such information is valuable for public health systems in developing countries that frequently underreport or misreport epidemics.

## Methods

### Collection of Death Data

The registrar of births and deaths (RBD) of Ahmedabad, who is a subordinate officer to the medical officer of health, registers all births and deaths within the city limits under the Registration of Births and Deaths Act. Deaths are registered in 2 ways. Deaths that occur in a hospital are reported by hospital authorities, who provide a medical certificate of death that is sent to the RBD officer in the city ward in which the hospital is located. Deaths that occur at home are reported by the family to the local RBD officer of the ward in which their home is located.

Deaths are compiled and sent from the RBD ward office to the RBD central office and subsequently communicated to the state level registrar of birth and death. Death data used in this study were provided by the medical officer of health of the city. Data include monthly total deaths registered in Ahmedabad during 2002–2006.

### Collection of Chikungunya Case Data

During the chikungunya epidemic, the city health department collected, compiled, and reported data on suspected cases of chikungunya from municipal hospitals and health centers. Data include monthly reported cases of chikungunya, blood samples sent for testing, and samples positive for chikungunya virus infection in Ahmedabad starting in April 2006. Few data were reported by private hospitals, dispensaries, and private practitioners in the city, who treat many patients.

### Statistical Analysis

Average mortality rate for each month during 2002–2005 (years before the epidemic) was calculated by dividing the average number of deaths for each month by the average population. Average mortality rate for each month in 2006 was calculated by dividing the number of registered deaths for each month by the monthly population. The expected number of monthly deaths for each month in 2006 was calculated by multiplying the average mortality rate for each month (2002–2005) by the monthly population in 2006. Because there were 12 estimates of expected deaths (1 for each month), we used the more conservative simultaneous confidence interval (CI) and the Bonferroni method (*9*) instead of a simple CI for each month separately. Excess deaths for each month in 2006 were the difference between actual observed number of deaths and expected number of deaths. Average monthly mortality rates for 2002–2005 were then compared with the mortality rate for 2006 (epidemic year).

## Results

The medical officer o fhealth in Ahmabadad reported 60,777 suspected chikungunya cases in 2006. The peak of the epidemic occurred in August and September 2006 when 55,593 (91.5%) of the cases were reported. A total of 84 (54.5%) of 154 blood samples tested were positive for chikungunya virus. Of these 84 confirmed chikungunya cases, 10 were fatal (case-fatality rate 11.9%).

A monthly distribution of cases of chikungunya, actual and expected number of deaths in 2006, and monthly average mortality rates for 2002–2005 and 2006 per 10,000 persons are shown in the Table. The number of deaths and mortality rates increased substantially from August through November 2006 compared with values for 2002–2005 for the same months. Mortality rates for August, September, and October 2006 increased 22%, 57%, and 33%, respectively, compared with average mortality rates for these months for 2002–2005. The highest numbers of chikungunya cases were also reported during these months. A total of 31,496 deaths were registered in 2006 compared with 28,440 (99% CI 27,500–29,380) expected deaths for the same year based on average number of deaths for the last 4 years. There were ≈3,056 additional deaths registered in Ahmedabad in 2006 compared with the expected number of deaths for 2006. A comparison of the monthly distribution of actual deaths in 2006 with expected deaths showed a rapid increase in deaths registered from August through November 2006. In these 5 months, 2,944 additional deaths (96.34% of total additional deaths for 2006) occurred when compared with the expected number of deaths for the same months for the previous 4 years. Excess number of deaths peaked in September 2006, when 1,448 additional deaths (47.38% of total additional deaths for 2006) occurred when compared with the expected deaths for September (Figure).

**Table:** Monthly chikungunya cases, deaths, and mortality rates, Ahmedabad, India, 2002–2005 and 2006*

Month	Chikungunya cases, 2006	Mortality rate/10,000 (99% CI), 2002–2005	Expected deaths, 2006 (99% CI)	Actual deaths, 2006	Excess deaths, 2006	Mortality rate/10,000, 2006	% Change in mortality rate
Jan	ND	6.19 (6.00–6.41)	2,422 (2,342–2502)	2,559	137	6.54	+5.66
Feb	ND	5.56 (5.37–5.76)	2,180 (2,105–2255)	2,227	47	5.68	+2.14
Mar	ND	5.76 (5.56–5.95)	2,264 (2,187–2,341)	2,337	73	5.95	+3.24
Apr	434	5.75 (5.53–5.92)	2,260 (2,183–2,337)	2,150	−110	5.47	−4.89
May	141	6.16 (5.93–6.33)	2,428 (2,349–2,507)	2,510	82	6.37	+3.37
Jun	31	5.80 (5.56–5.95)	2,290 (2,213–2,367)	2,156	−134	5.46	−5.86
Jul	184	5.50 (5.27–5.65)	2,177 (2,102–2,252)	2,270	93	5.73	+4.27
Aug	28,233	6.08 (5.82–6.21)	2,410 (2,331–2,489)	2,942	532	7.42	+22.09
Sep	27,360	6.40 (6.12–6.52)	2,541 (2,460–2,622)	3,989	1,448	10.05	+56.96
Oct	3,555	5.92 (5.64–6.03)	2,355 (2,277–2,433)	3,121	766	7.85	+32.51
Nov	539	6.27 (5.97–6.38)	2,500 (2,420–2,580)	2,698	198	6.77	+7.90
Dec	300	6.54 (6.22–6.63)	2,613 (2,531–2,695)	2,537	−76	6.35	−2.90
Total	60,777		28,440 (27,500–29,380)	31,496	3,056		

*CI, confidence interval; ND, no data available.

**Figure:**
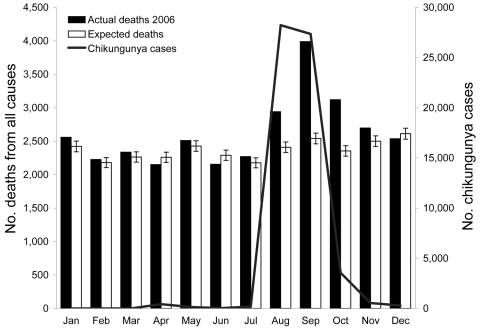
Monthly chikungunya cases, expected deaths, and reported deaths, Ahmedabad, India, 2006. Error bars show 99% confidence intervals. Jul–Dec, differences were statistically significant.

The temporal relationship between chikungunya cases and expected mortality rates and actual mortality rates in 2006 is shown in the Figure. The peak in chikungunya cases in August–September coincides with the peak in actual deaths in 2006.

## Discussion

Analysis of our data shows that the mortality rate in Ahmedabad increased substantially in 2006 when compared with rates for the previous 4 years. A total of 3,056 excess deaths occurred in 2006 (the epidemic year) when compared with the expected number of deaths for that year. A substantial increase in deaths reported was observed from August through November 2006 (2,944 excess deaths in these months). The number of reported chikungunya cases also showed a peak in August and September 2006, which coincided temporally with the peak in number of deaths in Ahmedabad (Figure).

The main issues of contention are whether these excess deaths were caused by chikungunya and whether such excess deaths will occur in future years without chikungunya epidemics. No major adverse event or other epidemic occurred in Ahmebabad in August–November 2006 other than the chikungunya epidemic. Our epidemiologic evidence shows that the epidemic is the most plausible explanation for the large increase in deaths in Ahmedabad in August–November 2006. However, other unidentified causes cannot be ruled out. Similar data from other cities and areas affected by the chikungunya epidemic may help establish the link between chikungunya and excess deaths.

There are 2 major problems with reporting of deaths in Ahmedabad. The cause of death is poorly reported, and the RBD does not separate death data for residents and nonresidents. Inclusion of patients from surrounding rural areas who died in city hospitals could have resulted in excess deaths being reported during the epidemic. However, this was a problem in years before the epidemic (2002–2005) as well. A review of deaths registered in rural areas outside the city limits of Ahmedabad showed no major decrease during the epidemic months of 2006 over previous years. Thus, the increase in number of deaths caused by migration of sick patients cannot explain this major increase in deaths in 2006, although this factor may have contributed to it.

An excess in total deaths was also reported for the chikungunya epidemic on Réunion Island during February–April 2006 (*10*). A total of 260 excess deaths were reported on Réunion Island during the epidemic, of which 254 were directly or indirectly attributed to chikungunya (mortality rate attributed to chikungunya 1/1,000) (*4*,*10*). Most of the excess deaths on Réunion Island were persons >75 years of age. Of 10 confirmed deaths in Ahmedabad caused by chikungunya, 2 were persons >80 years of age, 4 were persons 60–70 years of age, and 3 were persons <60 years of age.

The genomic sequences of chikungunya virus isolates from India were similar to that of a recent isolate from Réunion Island (*11*). Because of this finding, the mortality rate on Réunion Island can be applied to the epidemic in India to estimate the probable number of deaths that may have occurred. With limited case data reported from India and a mortality rate on Réunion Island of 1 per 1,000 cases, it was previously estimated that India would have ≈1,200–19,000 deaths caused by the chikungunya epidemic (*12*). The excess number of deaths observed during the epidemic in Ahmedabad suggests that estimates of deaths caused by chikungunya in India need to be revised.

Despite the increase in deaths in Ahmedabad and reports of suspected deaths caused by chikungunya in Kerala State, India (*13*), no systematic and comprehensive investigation of deaths in relation to this epidemic has been conducted by government authorities at the national or state level in India. The government of India has declared repeatedly in the parliament that “there are no deaths directly attributable to Chikungunya” in India (*6*). Although 10 deaths caused by chikungunya were reported by the medical officer of health in Ahmebadad, the website of the government of India continues to report “zero deaths” (*5*). Further investigations on the cause of excess deaths are urgently needed to conclusively establish that chikungunya was the cause of excess deaths in Ahmedabad. Given the possible association of deaths with the chikungunya fever epidemic in Ahmedabad, public health authorities should investigate such epidemics in other countries. These investigations will help determine whether the virus has increased in virulence, which may also pose a greater threat outside the Indian Ocean region. Such studies would help detect and control similar epidemics and help governments to provide adequate warnings to travelers to chikungunya-endemic countries.

We report an increase in mortality rates in Ahmedabad during August–November 2006 (when a chikungunya epidemic occurred in this city) compared with previous months in 2006 and the same months in the past 4 years. The highest number of chikungunya cases was also reported in August and September. The city had ≈2,944 additional deaths during August–November 2006. Epidemiologic evidence shows that the increase in deaths in Ahmedabad was largely attributable to the chikungunya epidemic. Given poor reporting of deaths, an unexplained cause of death cannot be ruled out. Mortality rate data for Ahmedabad are consistent with observations of other researchers that the virus may have mutated and become more dangerous than reported (*8*). Public health authorities must investigate recent epidemics. Otherwise, developing countries may not be able to detect and combat severe future epidemics of other reemerging diseases such as avian influenza and severe acute respiratory syndrome. If our findings are validated by studies in other regions of India and elsewhere, it would assist the international health community to be better prepared in dealing with future epidemics of emerging infectious diseases and reduce associated deaths.
